# Co-culture with embryonic renal stromal progenitor cell enhances podocyte differentiation and modulates doxorubicin-induced injury responses

**DOI:** 10.1016/j.bbrep.2026.102873

**Published:** 2026-11-06

**Authors:** Tien-Hsiang Tang, Kotaro Doi, Yasuyuki Sakai, Masaki Nishikawa

**Affiliations:** a Department of Chemical System Engineering, Graduate School of Engineering, The University of Tokyo, Tokyo, Japan; b Department of Nephrology and Endocrinology, The University of Tokyo Hospital, Tokyo, Japan

**Keywords:** Podocyte differentiation, Renal stromal progenitor cells, Stromal–podocyte co-culture, Glomerular doxorubicin-induced injury

## Abstract

Podocytes are specialized epithelial cells that play a central role in maintaining the selective permeability of the glomerular filtration barrier of the kidney. However, conventional podocyte mono-culture systems often lack the cellular microenvironment required to fully support podocyte differentiation. Renal stromal progenitor cells (rSPCs) contribute to glomerular development and give rise to stromal derivatives, including mesangial cells, suggesting that they may provide developmental cues that support podocyte differentiation. Therefore, this study investigates the effect of co-culturing heat-sensitive mouse podocytes (HSMPs) with mouse embryonic rSPCs on podocyte differentiation and function in a membrane-based system. Podocytes were cultured alone or in combination with sorted embryonic rSPCs on porous membranes of different pore sizes (0.4 μm and 8 μm). The results showed that co-culture with rSPCs on 8 μm membranes significantly enhanced podocyte marker expression and promoted podocyte-like morphology, likely due to improved cellular interactions. The co-culture model maintained size-selective permeability and exhibited a distinct dose-dependent response to doxorubicin-induced injury, suggesting that rSPCs modulate podocyte sensitivity to toxic stress. These findings suggest that embryonic rSPCs provide a supportive microenvironment that enhances podocyte differentiation and stability, offering a more physiologically relevant *in vitro* model for studying glomerular development and injury.

## Highlights

• A membrane-based monolayer coculture of podocytes and embryonic rSPCs was developed.
• rSPCs enhanced podocyte marker expression, particularly with 8 μm pores.
• rSPC coculture altered podocyte responses to doxorubicin-induced injury.
• The model enables *in vitro* studies of stromal–podocyte interactions.

## 1 Introduction

Podocytes are highly specialized epithelial cells that cover the outer surface of glomerular capillaries and play an essential role in maintaining the glomerular filtration barrier [1]. Together with glomerular endothelial cells and the glomerular basement membrane, podocytes contribute to the selective permeability of the glomerulus, allowing water and small solutes to pass while restricting the leakage of macromolecules such as plasma proteins [2], [3]. Podocyte function depends on their unique cellular architecture, including interdigitating foot processes, slit diaphragm-associated proteins, and a highly organized actin cytoskeleton. Therefore, podocyte injury, dedifferentiation, or loss is closely associated with glomerular barrier dysfunction and proteinuric kidney diseases [4], [5].

*In vitro* podocyte models are important tools for studying glomerular development, filtration function, and drug-induced podocyte injury. Conditionally immortalized podocyte cell lines have been widely used because they can be expanded under permissive conditions and induced to differentiate under non-permissive conditions [6], [7]. In addition, advances in pluripotent stem cell differentiation have enabled the generation of podocyte-like cells and kidney organoids that partially recapitulate nephrogenesis. Various protocols demonstrated that human pluripotent stem cells (hiPS) can be directed toward nephron progenitor (NPC) and kidney organoid lineages, providing useful platforms for developmental studies, disease modeling, and drug screening [8], [9], [10]. However, despite these advances, many current *in vitro* podocyte models still have limitations, including incomplete maturation, insufficient cellular interactions, and difficulty reproducing the multicellular microenvironment of the developing glomerulus. Kidney organoids also frequently exhibit immature cell states and a limited representation of supporting vascular and stromal components, which may limit their ability to fully reproduce glomerular structure and function [11].

During kidney development, nephron formation is regulated by coordinated interactions among NPCs, ureteric bud (UB)-derived epithelial structures, endothelial cells, and renal stromal progenitor cells (rSPCs). Although NPCs give rise to glomerular epithelial lineages, including podocytes, the surrounding renal stroma provides important structural and signaling support during nephrogenesis. Recent reviews have emphasized that rSPCs are not merely passive supporting cells, but actively contribute to kidney morphogenesis, nephron patterning, and the formation of specialized stromal derivatives [12], [13]. In particular, rSPCs can give rise to functionally important renal stromal lineages, including mesangial cells and renin-producing cells. Mesangial cells are especially relevant to glomerular development because they interact with podocytes and endothelial cells to support the formation and organization of the glomerular capillary tuft [14], [15].

The importance of stromal contribution to glomerular development is further supported by studies showing that stromal signaling pathways regulate mesangial cell formation and glomerular structure. For example, Mesangial cells are specialized stromal derivatives within the glomerular tuft, and Notch signaling in rSPCs has been reported to be required for mesangial cell formation during mouse kidney development [16]. In addition, studies using reconstructed kidney organoid systems have shown that the combination of NPCs, UB, and rSPCs is important for generating more organotypic kidney structures, suggesting that rSPCs provide developmental cues that cannot be fully replaced by epithelial cells alone [17], [18]. These findings suggest that incorporating embryonic rSPCs into podocyte culture systems may provide a more developmentally relevant microenvironment for podocyte differentiation.

In this study, we investigated whether embryonic rSPCs enhance podocyte differentiation in a membrane-based co-culture system. rSPCs were selected instead of mature mesangial cells because they may retain developmental signaling and extracellular matrix-remodeling properties that more closely reflect the stromal microenvironment of glomerulogenesis. Furthermore, while rSPCs have been incorporated into organoid and reconstructed kidney models, their use in podocyte monolayer co-culture systems suitable for filtration-related analysis remains limited. We therefore established a porous membrane-based HSMP–rSPC co-culture model to evaluate podocyte differentiation, stromal–podocyte interactions, size-dependent permeability, and responses to doxorubicin-induced injury.

## 2 Materials and methods

### 2.1 Animals

Pregnant female C57BL/6JJcl mice were purchased from CLEA Japan Inc. and housed in the animal facility. All animal procedures were conducted in accordance with the Act on Welfare and Management of Animals of Japan, the Fundamental Guidelines for Proper Conduct of Animal Experiments and Related Activities in Academic Research Institutions issued by the Ministry of Education, Culture, Sports, Science and Technology, the Guidelines for Proper Conduct of Animal Experiments issued by the Science Council of Japan, and the University of Tokyo Rules concerning Animal Experiments. The experimental protocol was approved by the relevant committee of the University of Tokyo (Approval Number: A2024E024-01). The study is reported in accordance with the ARRIVE guidelines.

### 2.2 Cell culture

Heat-sensitive mouse podocytes (HSMPs) [19] were kindly gifted by the Shankland Lab at the University of Washington. For cell propagation, HSMPs were cultured at 33°C in a humidified incubator with 5% CO_2_ in RPMI-1640 medium (FUJIFILM Wako, Japan) supplemented with 10% fetal bovine serum (FBS; Biowest, France), 10 mmol/L HEPES buffer (FUJIFILM Wako, Japan), 1 mmol/L sodium pyruvate (FUJIFILM Wako, Japan), 50 U/mL murine interferon-γ (IFN-γ; PeproTech, USA), 1% penicillin/streptomycin (FUJIFILM Wako, Japan). Cells were passaged using Accutase (Innovative Cell Technologies, USA) for dissociation, and the culture medium was refreshed every two days. For differentiation, HSMPs were seeded onto the apical side of 24-well porous membrane inserts at a density of 1.25 × 10^5^ cells/cm^2^ to achieve high confluence at the start of differentiation. Inserts with 0.4 μm porous membranes were obtained from Greiner Bio-One, and 8 μm porous membranes were obtained from Sterlitech. The approximate culture surface area was 0.33 cm^2^. Before seeding, membranes were coated with 1 μg/cm^2^ laminin-521 (Corning, USA) and 5 μg/cm^2^ collagen type IV (Nitta Gelatin, Japan). Differentiation was induced by culturing the cells at 37°C in a humidified 5% CO_2_ incubator for 14 days in the absence of IFN-γ.

### 2.3 Building a co-culture system with mouse-derived rSPCs

Embryonic kidneys were isolated from C57BL/6JJcl mice at embryonic day 18 (E18) in DMEM/F-12 supplemented with 1% insulin-transferrin-selenium (ITS; Thermo Fisher Scientific, USA), 1% penicillin/streptomycin. The sex of the embryos was not determined; therefore, sex-specific effects were not evaluated in this study. Isolated kidneys were minced into small pieces using dissecting scissors, washed with cold phosphate-buffered saline (PBS; FUJIFILM Wako, Japan), and enzymatically dissociated with 2 mg/mL collagenase (FUJIFILM Wako, Japan) in a 37°C water bath for 9 min. During digestion, the tissue suspension was gently pipetted for 30 s every 3 min to promote uniform dissociation. The reaction was stopped by adding an equal volume of FBS, and the resulting cell suspension was filtered through a 70 μm cell strainer (pluriSelect Life Science, USA). After centrifugation, the cell pellet was resuspended in dissection medium.

For cell sorting, dissociated cells were incubated on ice for 30 min with biotinylated mouse integrin α8 antibody (ITGA8; R&D Systems, USA) and APC-conjugated anti-mouse CD140a antibody (PDGFRα; BioLegend, USA). Integrin α8 staining was detected using an Alexa Fluor 488-conjugated anti-goat secondary antibody (Abcam, UK). Cell sorting was performed using a Cell Sorter SH800S (Sony, Japan), and the acquired data were analyzed using FlowJo software (TreeStar Inc.).

Sorted rSPCs were seeded onto the basal side of 0.4 or 8 μm porous membrane culture inserts at a density of 4 × 10^4^ cells/well. Before seeding, membranes were coated with iMatrix-511 (Matrixome, Japan) at a 1:100 dilution in PBS. After 4 h of incubation to allow cell attachment, the inserts were turned back to the standard orientation, and HSMPs were seeded onto the apical side at a density of 1.25 × 10^5^ cells/cm^2^. The co-culture system was maintained in HSMP differentiation medium, which was refreshed every 2 days. Cells were cultured for 14 days, and analyses were performed after the differentiation period. In this co-culture model, the apical side with podocytes represents the urinary space, while the basal side with rSPCs represents the capillary lumen to simulate an in vivo glomerulus (Fig. 1A).

**Fig. 1:**
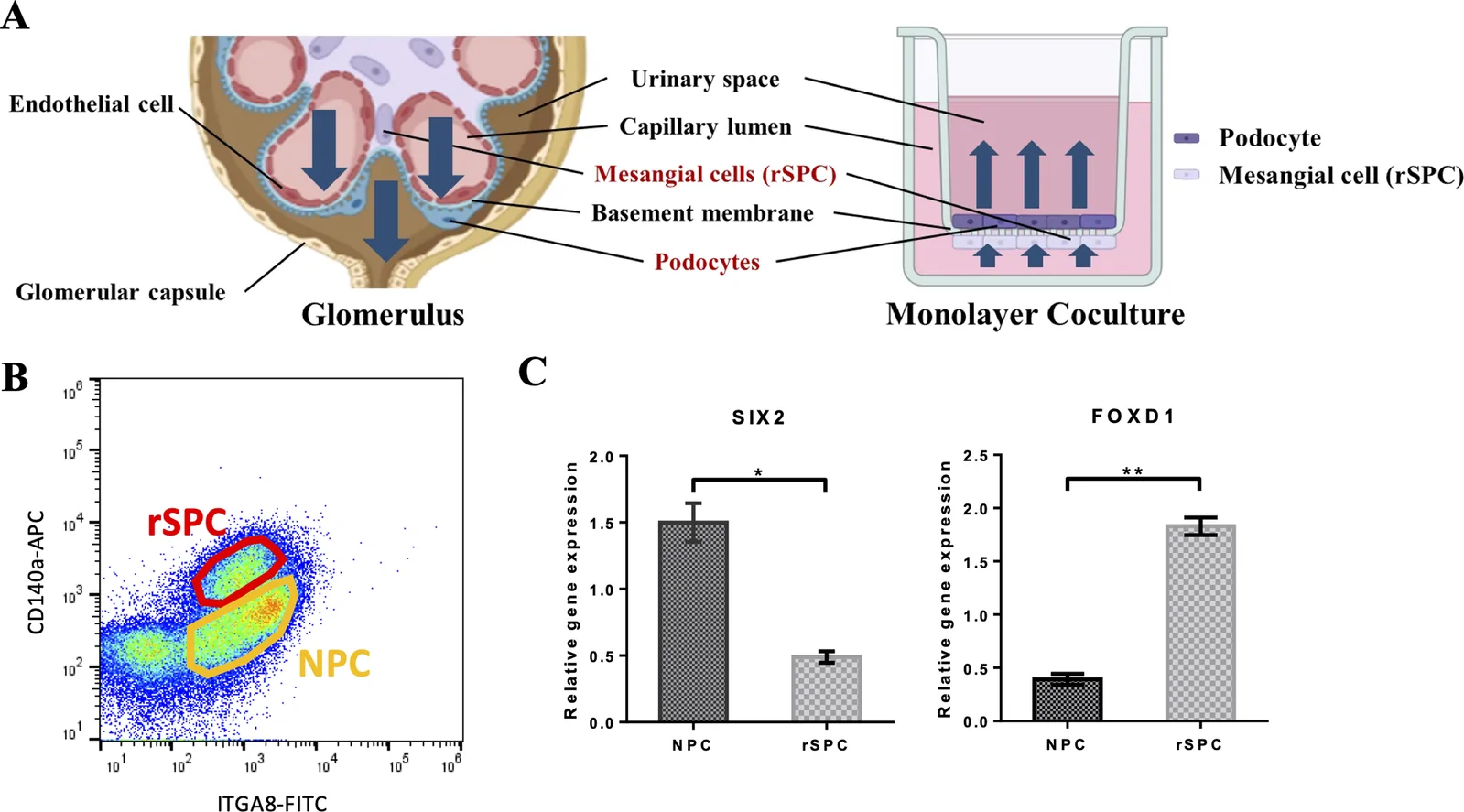
Construction of the glomerular co-culture model of rSPCs and **podocytes.** (A) A comparison between the structure of a glomerulus and the rSPCs-podocyte co-culture model. (B) Flow cytometry analysis of E18.5 mouse embryonic kidneys with anti-ITGA8 and anti-CD140a antibodies. rSPCs were sorted as CD140+ population, which was red-gated. (C) Relative expression levels of NPCs- and rSPCs-associated genes to GAPDH expression (n = 3). All data was calculated by 2^−ΔΔCt^. *P < 0.05, **P < 0.01, *t*-test.

### 2.4 Quantitative PCR (qPCR)

After removing rSPCs from the basal side with a cell scraper, total RNA was extracted separately using TRIzol reagent (Thermo Fisher Scientific, USA) and reverse-transcribed using PrimeScript RT Master Mix (Takara Bio, Japan) according to the manufacturer's instructions. Quantitative real-time PCR was performed using the StepOnePlus Real-Time PCR System (Thermo Fisher Scientific, USA). Gene expression levels were normalized to GAPDH using the Ct values. The primer sequences used in this study are listed in Table 1.

**Table 1:** Primer sequences.

Name	Forward/Reverse	Sequence (5′ to 3′)
GAPDH	F	AGGTCGGTGTGAACGGATTTG
	R	TGTAGACCATGTCGTTGAGGTCA
SIX2	F	CAAGTCAGCAACTGGTTCAAGA
	R	ACTGCCATTGAGCGAGGA
FOXD1	F	TTCGGATTCTTGGACCAGAC
	R	CAAGTCAGGGTTGCAGCATA
NPHS1	F	AATTGGCGGCTGGGTCTTAG
	R	TTTGTGTGGCATACACTGTCAGG
PODX1	F	TCCTTGTTGCTGCCCT
	R	TTCCAAGGTTGGGTTG
WT1	F	TGAAGACCCACACCAGGACTC
	R	TGTGATGGCGGACCAATTC
SYNPO	F	TCCTCACCTAATGCCACACTC
	R	GCTGGAGGGTTTTGGTTGATA
ACTA2	F	CCTTCGTGACTACTGCCGAG
	R	TATAGGTGGTTTCGTCCATCGCC
PDGFRB	F	CCATGGGTGGAGATTCACAG
	R	TCCATCGGATCTCATAGCGTG

### 2.5 Immunofluorescence imaging

Cells were fixed with 4% paraformaldehyde (PFA; Sigma-Aldrich, USA) at room temperature for 15 min and washed three times with PBS. For permeabilization, samples were incubated in PBS containing 0.1% Triton X-100 (Sigma-Aldrich, USA) and 0.2% Tween-20 (FUJIFILM Wako, Japan) at room temperature for 15 min. Blocking was performed with 3% bovine serum albumin (BSA; FUJIFILM Wako, Japan) in PBS-T at room temperature for 1 h.

Primary antibodies were diluted in 3% BSA in PBS, and the following antibodies were used: anti-synaptopodin antibody (SYNPO; 1:200; Santa Cruz Biotechnology, USA), anti-Wilms tumor protein antibody (WT1; 1:300; Abcam, USA), and anti-alpha-smooth muscle actin antibody (ACTA2; 1:200; Novus Biologicals, USA). Samples were incubated with primary antibodies overnight at 4°C. The corresponding secondary antibodies, diluted in 3% BSA solution, were added, and the samples were incubated at room temperature for 1 h. F-actin was stained with Acti-stain 488 (Cytoskeleton, USA) at room temperature for 1 h according to the manufacturer's instructions. Nuclei were counterstained with DAPI (1:1000; DOJINDO, Japan) at room temperature for 15 min. Immunofluorescence images were acquired using a confocal microscope (Olympus, Japan) and analyzed with ImageJ.

### 2.6 Scanning electron microscope (SEM)

Cells were fixed with 2.5% glutaraldehyde (MP Biomedicals, USA) at 4°C overnight. Dehydration was then performed through a graded ethanol series by sequential incubation in 30%, 50%, 70%, and 100% ethanol. After the final dehydration step, samples were allowed to air-dry completely in a chemical fume hood. The dried samples were then observed using a TM3030 Miniscope scanning electron microscope (Hitachi, Japan), and SEM images were acquired for morphological analysis.

### 2.7 Size-dependent permeability of the glomerular barrier

On the final day of co-culture, FITC-dextrans of different molecular weights (4, 40, and 70 kDa; Sigma-Aldrich, USA) were added to the medium in the basal compartment of the Transwell system at an initial concentration of 0.1 mg/mL. The samples were then incubated at 37°C for 24 h to evaluate dextran permeability across the cell-seeded porous membranes. After incubation, medium from the apical and basal compartments was collected separately. Fluorescence intensity was measured using a microplate reader (Agilent BioTek Synergy H1) and used to assess the diffusion of FITC-dextran from the basal to the apical side.

### 2.8 Toxicological reactivity to doxorubicin

To evaluate the toxicological response of podocytes to doxorubicin, differentiated HSMP mono-culture and HSMP/SP co-culture samples were treated with doxorubicin hydrochloride (FUJIFILM Wako, Japan) at different concentrations. Doxorubicin was diluted in differentiation medium and added to the basal side of the culture system on the final day of differentiation. After treatments, cells were incubated at 37°C in a humidified 5% CO_2_ incubator for 24 h. Basal administration was selected to simulate the exposure from the capillary-facing side of the glomerular filtration barrier. However, because doxorubicin concentrations in the apical compartment were not directly measured, the reported concentrations represent the initial concentrations applied to the basal compartment rather than the exact concentrations experienced by the podocytes.

Following doxorubicin exposure, cell viability was evaluated using Calcein-AM staining. Briefly, cells were incubated with Calcein-AM solution at 37°C, and fluorescence images were acquired using a confocal microscope. The Calcein-AM-positive area was quantified using ImageJ and used as an indicator of viable cell area. The viable cell area in each treatment group was normalized to that of the untreated control, which was defined as 100%. Dose–response curves were generated by plotting the normalized viable area against the logarithm of the doxorubicin concentration. IC_50_ values were estimated in GraphPad Prism using nonlinear regression with a four-parameter logistic model (log[inhibitor] versus normalized response, variable slope).

For morphological evaluation of doxorubicin-induced injury, treated samples were fixed and subjected to immunofluorescence staining. Podocyte injury and cytoskeletal organization were assessed using SYNPO and F-actin staining, while ACTA2 staining was used to identify stromal-lineage cells in the co-culture condition. Images were acquired using a confocal microscope and analyzed to compare the response of mono-culture and co-culture models to doxorubicin-induced toxicity.

### 2.9 Data analysis

All quantitative data are presented as the mean ± standard deviation (SD). Statistical analyses were performed using GraphPad Prism software. Comparisons between two groups were conducted using Student's t-test. Differences were considered statistically significant when p < 0.05. Significance levels are indicated in the figures as follows: *p < 0.05, **p < 0.01, and ***p < 0.001.

## 3 Results

### 3.1 rSPC sorting

We sorted embryonic renal cells by using ITGA8 and PDGFRα antibodies. NPCs could be identified as ITGA8^+^/PDGFRα^−^ population (yellow). In contrast, PDGFRα^+^ cells (red) will be identified as rSPCs (Fig. 1B). We analyzed sorting efficiency by measuring the expression levels of *Six2*, an NPC marker, and *Foxd1*, an rSPC marker, using qPCR. Samples were compared to cells that were before sorting, and the relative gene expression levels were equal to 1 in each gene. The results show that the expression level of *Six2* is much higher in NPC samples, while that of *Foxd1* is higher in rSPC samples (Fig. 1C). Thus, we confirmed that both cell types were sorted into the correct populations using this method.

### 3.2 Effects of co-culture with rSPC on gene expression

Membranes with two different pore sizes, 0.4 and 8 μm, were used to evaluate the effect of rSPC co-culture on HSMP differentiation. First, HSMP cells were co-cultured with rSPCs on 0.4 μm porous membranes, and the expression of podocyte-associated markers, including *Nphs1, Wt1, Podxl, and Synpo*, was analyzed by qPCR. Gene expression levels were converted to a log scale, and undifferentiated HSMP cells before the induction phase were used as the control, with their relative expression level set to 1. Under the 0.4 μm membrane condition, *Wt1* expression showed a slight increase in the co-culture group compared with the mono-culture group (Fig. 2A). However, *Nphs1* and *Synpo* expression showed no clear difference, while *Podxl* expression were slightly decreased in the co-culture condition.

**Fig. 2:**
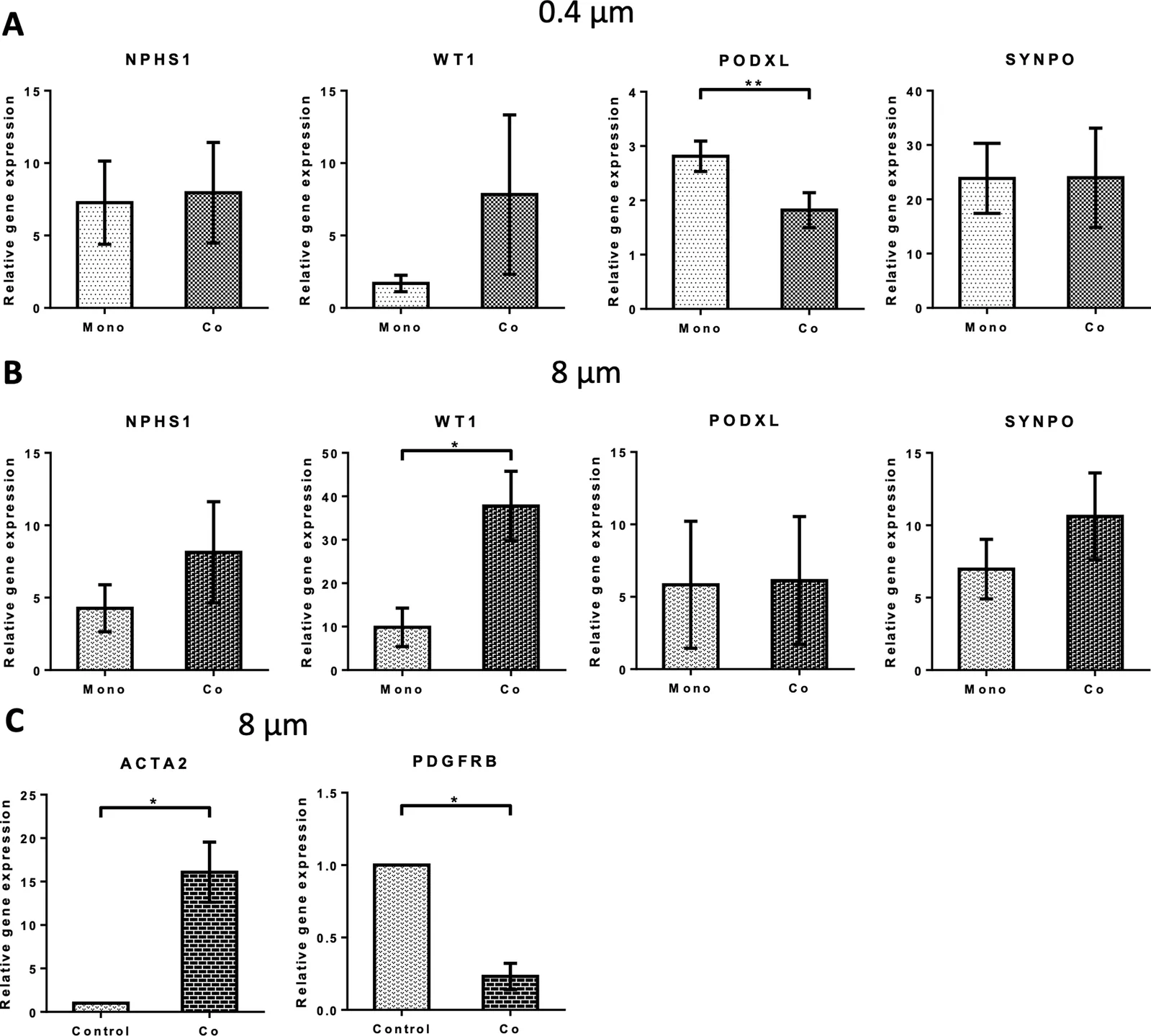
Comparison of podocytes under mono-culture and co-culture conditions on insert membranes with different p**ore sizes.** (A) Relative expression levels of podocyte-associated genes to GAPDH expression on 0.4 μm porous membranes (n = 3). All data was calculated by 2^−ΔΔCt^. *P < 0.05, **P < 0.01, *t*-test. (B) Relative expression levels of podocyte-associated genes to GAPDH expression on 8 μm porous membranes (n = 4). All data was calculated by 2^−ΔΔCt^. *P < 0.05, *t*-test. (C) Relative expression levels of stromal differentiation-associated genes to GAPDH expression on 8 μm porous membranes(n = 3). All data was calculated by 2^−ΔΔCt^. *P < 0.05, **P < 0.01, *t*-test.

Next, the same experiment was performed using 8 μm porous membranes. Interestingly, most podocyte-associated markers were upregulated in the co-culture condition, except *Podxl* (Fig. 2B). In particular, *Wt1* expression was significantly increased. At the same time, *Nphs1* and *Synpo* showed slight increases in the co-culture group compared with the mono-culture group. These results suggest that co-culture with rSPCs on 8 μm porous membranes may promote the differentiation or maturation of HSMP-derived podocytes.

We also examined the expression of stromal differentiation markers, *Acta2* and *Pdgfrb*, in SP cells. Freshly sorted rSPCs before culture were used as the control. After co-culture, *Acta2* expression was significantly upregulated. In contrast, *Pdgfrb* expression was downregulated (Fig. 2C). These results suggest that rSPCs underwent partial stromal differentiation during co-culture, although the specific stromal cell subtype could not be clearly determined based on these markers alone. Overall, the 8 μm porous membrane co-culture system appeared to better support interactions between HSMP and rSPCs, resulting in enhanced podocyte marker expression and partially reproducing a glomerular-like developmental microenvironment.

### 3.3 Effects of co-culture with rSPC on protein marker expression

We further examined the expression of podocyte-associated markers SYNPO and WT1, which were upregulated in the qPCR analysis. ACTA2 was also used as a stromal cell marker to evaluate the presence and distribution of stromal-lineage cells in the co-culture system. In the immunofluorescence images, podocytes cultured on 8 μm porous membranes showed stronger SYNPO signals together with clear WT1-positive cells, suggesting that co-culture with rSPCs may promote podocyte differentiation or maturation (Fig. 3). In the same condition, ACTA2-positive cells were clearly observed, indicating the presence of stromal/mesangial-like cells. The merged images further showed that WT1-positive cells were located near or within ACTA2-positive areas, suggesting possible spatial interaction between podocyte-lineage cells and stromal cells.

**Fig. 3:**
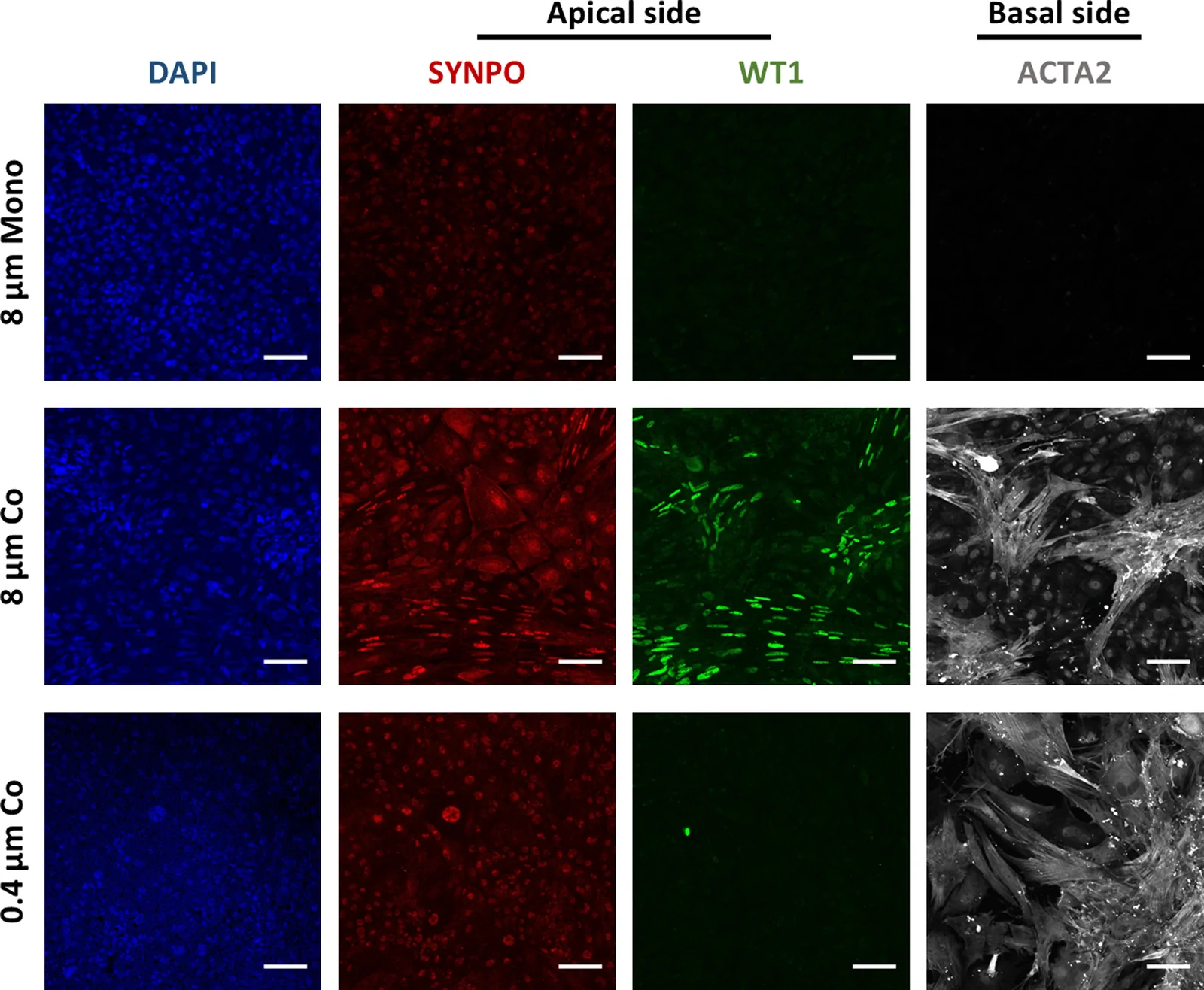
Investigation of pore size-dependent enhancements of podocyt**e markers.** Immunofluorescence staining of WT1 and SYNPO for podocyte markers and Acta2 for stromal markers in mono-culture and co-culture conditions. SYNPO and WT1 images were acquired from the apical side of the membrane to evaluate podocyte-lineage HSMPs, whereas ACTA2 images were acquired from the basal side to identify stromal-lineage rSPCs. Merged images are not shown because podocyte-marker and ACTA2 images were acquired from different z-positions corresponding to opposite sides of the membrane. Scale bar = 100 μm.

In contrast, under the 0.4 μm porous membrane condition, SYNPO-positive cells were still detected, but WT1 expression appeared weaker, suggesting that limited physical interaction between the two cell types may reduce the supportive effect of rSPCs on podocyte differentiation. To further investigate cell–cell interactions under the 8 μm porous membrane condition, SEM observations were performed. In the 0.4 μm membrane condition, the pores were too small to allow obvious cellular protrusions, and cells appeared to spread over and cover the membrane surface (Fig. 4A). In contrast, the 8 μm membrane contained pores large enough to permit cellular protrusion-like structures extending into or through the pores (Fig. 4B). The protrusion-like structures observed in the 8 μm membrane were larger than the 0.4 μm pores, suggesting that similar protrusive interactions would be physically restricted in the 0.4 μm membrane condition. These findings support the idea that increased cellular interaction through larger membrane pores enhances podocyte differentiation. Overall, these results suggest that stromal cell co-culture on 8 μm porous membranes enhances podocyte marker expression and supports podocyte-like differentiation.

**Fig. 4:**
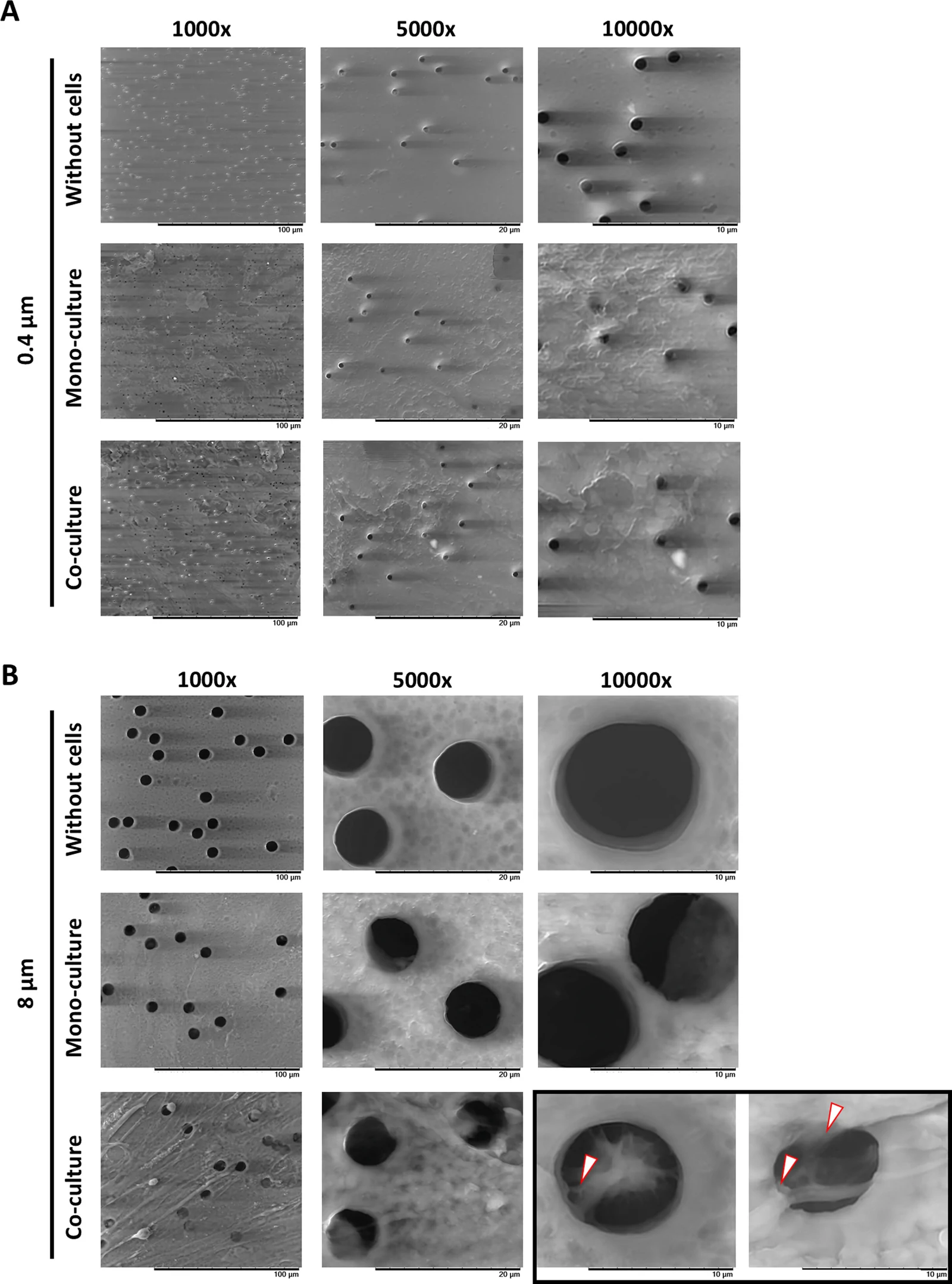
SEM observation of cell–membrane morphology in 0.4 and 8 μm porous **membranes.** (A) 0.4 μm porous membrane without cells, mono-culture and co-culture under 1000x, 5000x, and 10000x magnification. Scale bar = 100, 20, and 10 μm. (B) 8 μm porous membrane without cells, mono-culture and co-culture under 1000x, 5000x, and 10000x magnification. Scale bar = 100, 20, and 10 μm. Arrows indicate cellular extensions through a 8 μm pore (dark area).

### 3.4 Analysis of podocyte barrier function

Dextran of different molecular sizes was used to analyze the size-dependent permeability of the podocyte barrier. In human bodies, small molecules (4 kDa) are permitted to pass through, whereas large ones (40 and 70 kDa) are restricted in the bloodstream. Dextran was added to the basal side of the transwell membranes to evaluate diffusion and permeability across the membrane. First, empty membranes were tested with dextran of different molecular sizes to confirm whether 24 h was sufficient for diffusion. After 24 h, the dextran concentrations in the upper and basal compartments became nearly equivalent, indicating that dextran could diffuse sufficiently through the membrane and that the assay duration was appropriate for evaluating permeability performance (Fig. 5A).

**Fig. 5:**
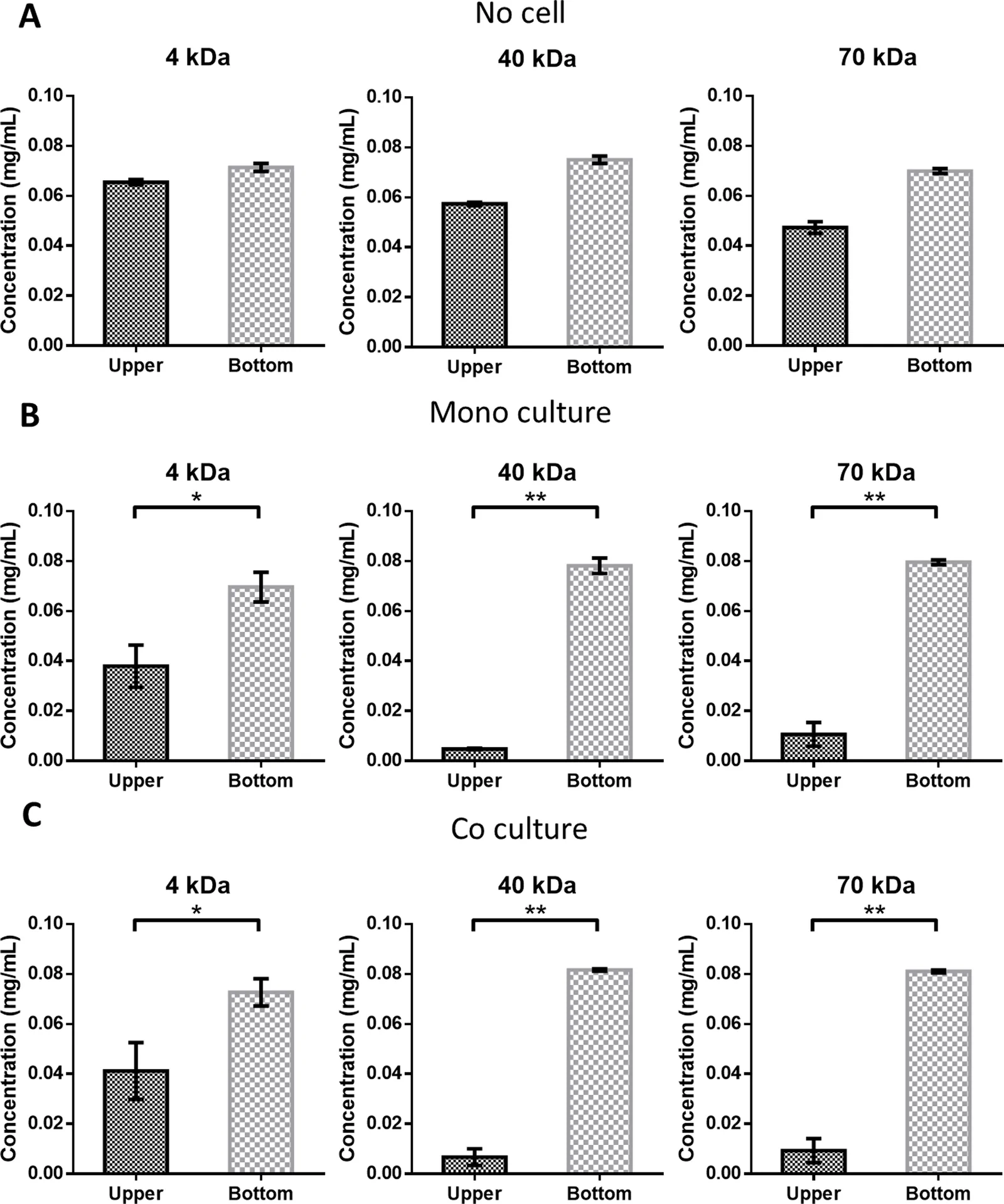
Evaluation of the selective permeability of the glomerulus using dextran. (A) Permeability of empty 8 μm porous membranes (n = 4). (B) Size-dependent filtration of mono-cultured podocytes (n = 3). *P < 0.05, **P < 0.01, *t*-test. (C) Size-dependent filtration of co-cultured podocytes with SPs (n = 3). *P < 0.05, **P < 0.01, *t*-test.

Next, the same assay was performed using membranes seeded with podocytes under mono-culture or co-culture conditions to evaluate size-dependent permeability function. In both groups, the smaller molecule, 4 kDa dextran, passed through the cell layer, whereas larger molecules, 40 kDa and 70 kDa dextran, were more strongly restricted (Fig. 5B and C). Although the co-culture condition did not show a clear difference compared with the mono-culture condition, the co-culture model still maintained size-dependent permeability, allowing smaller molecules to pass while limiting the passage of larger molecules. This result suggests that the co-culture system preserved the size-dependent permeability property of the podocyte layer, but did not significantly improve permeability compared with monoculture.

### 3.5 Dose-dependent doxorubicin-induced injury in podocytes

To evaluate the effects of SP cell co-culture on podocytes in doxorubicin-induced injury, mono-culture and co-culture models were treated with increasing concentrations of doxorubicin and analyzed by immunofluorescence staining. In the mono-culture condition, doxorubicin treatment caused clear disruption of the podocyte layer, with reduced cell density, weaker SYNPO expression, and disorganized F-actin structure (Fig. 6A). In contrast, the co-culture condition showed better preservation of the cell layer after doxorubicin treatment. SYNPO-positive cells and F-actin organization were more clearly maintained compared with mono-culture, suggesting that co-culture with SP cells may reduce doxorubicin-induced podocyte injury. ACTA2-positive stromal/mesangial-like cells were also observed in the co-culture model, confirming the presence of stromal-lineage cells during the injury assay. These cells may contribute to the maintenance of podocyte structure under toxic conditions.

**Fig. 6:**
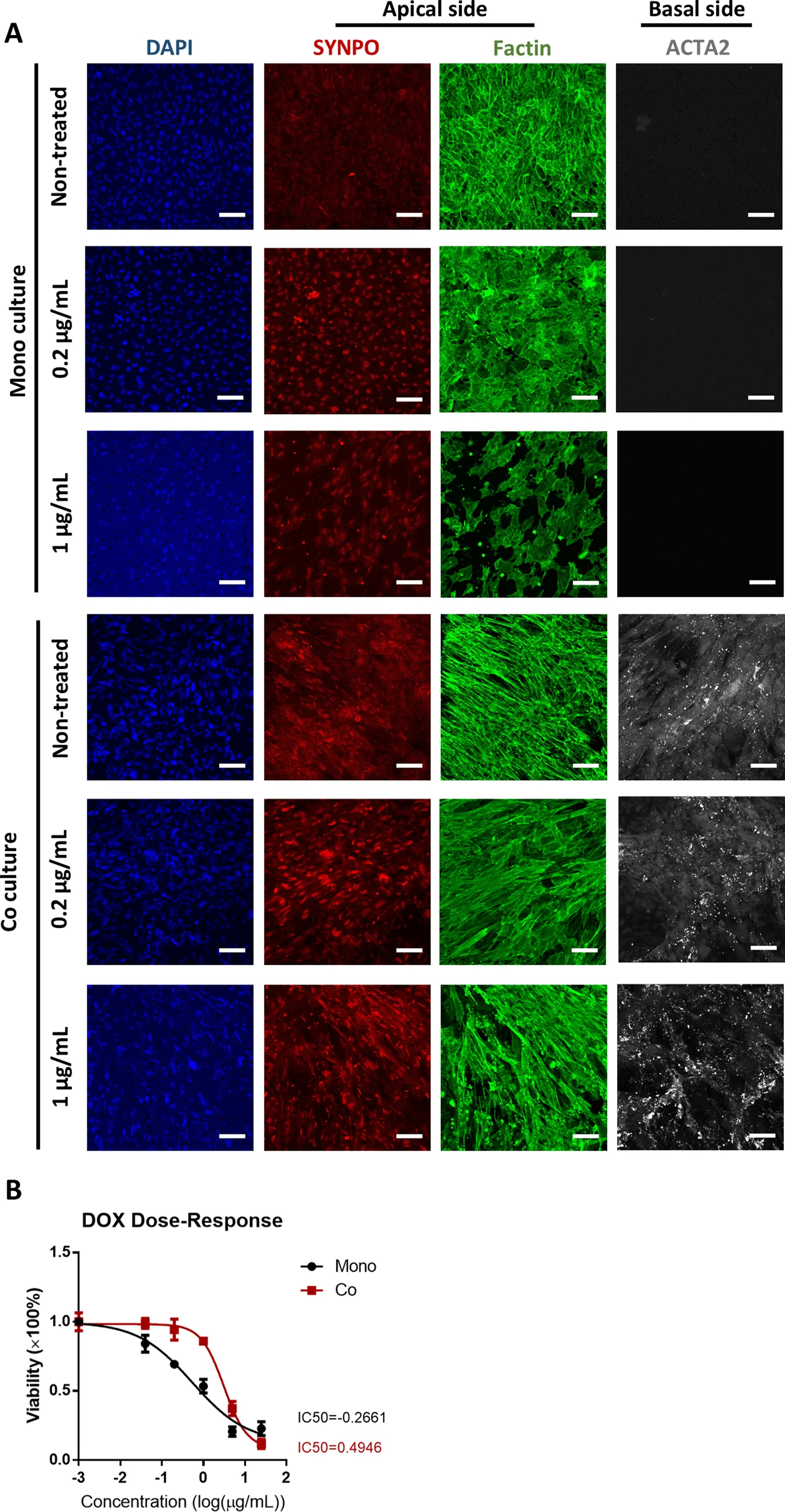
Doxorubicin-induced toxicity in podocytes under mono-culture and co-culture c**onditions.** (A) Immunofluorescence staining of SYNPO for podocyte marker, Factin for podocyte cytoskeleton, and Acta2 for stromal cells in mono-culture and co-culture conditions. 0, 0.2, and 1 μg/mL of doxorubicin were tested to investigate the differences between both culture conditions under injury. Scale bar = 100 μm. (B) Cell viability (%) of podocyte treated with various concentrations of doxorubicin evaluated by positive CalceinAM surface area (n = 3). IC_50_ of mono-culture and co-culture were calculated.

Cell viability was quantified by the surface area of Calcein-AM-positive signals, and doxorubicin concentrations were log-transformed for IC50 analysis. The dose–response curve showed that podocyte viability decreased more rapidly at lower doxorubicin concentrations in mono-culture than in co-culture, suggesting that co-culture with SP cells may maodulate early doxorubicin-induced injury (Fig. 6B).

Overall, compared with mono-culture, the co-culture model appeared to show greater resistance to doxorubicin-induced damage, as indicated by better preservation of podocyte marker expression and cytoskeletal organization. These results suggest that rSPC co-culture may modulate the microenvironment for podocytes and enhance the stability of the podocyte layer under drug-induced injury.

## 4 Discussion

In this study, we investigated whether embryonic rSPCs could support podocyte differentiation in a membrane-based co-culture system and evaluated doxorubicin-induced injury response. The qPCR results showed that co-culture on 8 μm porous membranes upregulated several podocyte-associated markers, particularly *Wt1*, and also showed slight increases in *Nphs1* and *Synpo*. Immunofluorescence staining further supported this tendency, as stronger SYNPO signals and clearer WT1-positive cells were observed in the co-culture condition compared with mono-culture. In contrast, the effect was weaker when 0.4 μm porous membranes were used. These results suggest that the membrane pore size is important for enabling closer interaction between podocytes and SP cells. That direct or near-direct communication between the two cell types may contribute to podocyte differentiation.

One possible explanation for the upregulation of podocyte markers is that embryonic rSPCs provide developmental signals that are normally present during glomerulogenesis. During kidney development, NPCs, rSPCs, endothelial cells, and UB-derived structures interact closely to regulate nephron patterning and glomerular formation. Renal stromal cells are not only structural supporting cells but also active regulators of nephrogenesis through signaling pathways such as WNT, BMP, FGF, Notch, and PDGF-related signaling. These pathways are known to participate in the maintenance, differentiation, and glomerular development of renal progenitors [12], [20].

Among the podocyte markers analyzed in this study, WT1 showed the clearest response to rSPC co-culture. WT1 is a key transcription factor involved in kidney development, NPC regulation, and podocyte identity. It is also essential for glomerular epithelial cell differentiation and maintenance [21], [22]. Importantly, previous studies have shown that WT1 reduction or dysfunction leads to decreased expression of podocyte-associated genes, including NPHS1 and PODXL, suggesting that these genes may act downstream of WT1-dependent transcriptional regulation [23]. Therefore, the marked increase in WT1 observed in the 8 μm co-culture condition may represent an important upstream change that contributes to the accompanying upregulation of podocyte differentiation markers, including NPHS1 and SYNPO.

However, because only marker expression was analyzed, the exact mechanism cannot be concluded from this study. Since soluble factors are expected to diffuse through both 0.4 and 8 μm membranes, the stronger effect observed with 8 μm membranes is unlikely to be explained by bulk diffusion alone. Instead, rSPCs may enhance podocyte marker expression through localized extracellular matrix remodeling, matrix-bound or short-range paracrine signals, or contact-dependent cues enabled by the larger pores and higher estimated open area. The SEM image showing cell protrusions extending through the pores supports the possibility that larger membrane pores allowed closer cell–cell communication. Further studies using pre-labeled rSPCs, conditioned medium, and extracellular matrix marker analysis are required to identify the responsible mechanism.

The stromal marker results also provide important information. *Acta2* was significantly upregulated in the co-culture system, whereas *Pdgfrb* was downregulated. *Acta2* expression suggests that rSPCs may have differentiated toward a stromal or mesangial-like phenotype. However, because *Pdgfrb* did not increase, the differentiation state of rSPCs remains unclear. Mesangial cell development is regulated by specific developmental pathways, including Notch signaling in rSPCs, which has been reported to be required for mesangial cell formation during mouse kidney development [16]. Therefore, although our co-culture system may partially reproduce a stromal-supported glomerular microenvironment, it cannot yet be concluded that the rSPCs differentiated into mature or correctly specified mesangial cells.

The doxorubicin injury experiment further demonstrated that mono-culture and co-culture systems respond differently to drug-induced stress. Based on Calcein-AM-positive area, viability in mono-culture decreased more rapidly at lower doxorubicin concentrations, whereas co-culture showed a delayed reduction in viability. However, because doxorubicin was applied only to the basal compartment to model vascular-side exposure, the observed difference may reflect not only altered podocyte sensitivity but also changes in drug diffusion, uptake, or retention by rSPCs before doxorubicin reached the apical podocyte layer. Therefore, these results should be interpreted as a distinct dose-dependent response of the coculture system under directional basal-side exposure, rather than direct evidence of uniform podocyte protection. Although direct evidence that rSPCs protect podocytes from doxorubicin-induced injury remains limited, previous studies using mesenchymal stromal cells have reported protective effects on podocytes through paracrine mechanisms, including VEGF-mediated pro-survival signaling [24], [25]. Since rSPCs are developmentally related to renal stromal and mesangial lineages, they may create a kidney-specific stromal niche that modifies podocyte injury responses. Nevertheless, stromal–podocyte interactions are likely context- and density-dependent, as excessive mesangial-lineage proliferation and extracellular matrix accumulation can contribute to glomerulosclerosis and secondary podocyte injury [26], [27]. Thus, rSPCs appear to modulate rather than uniformly protect against doxorubicin-induced podocyte injury.

Several limitations of this study should be addressed in future work. First, HSMPs are conditionally immortalized podocyte cell lines and may not fully recapitulate the phenotype of mature podocytes in vivo; therefore, the findings should be validated using more mature podocyte types. Secondly, although embryonic rSPCs expressed stromal-lineage markers, their differentiation toward a mesangial lineage was not specifically confirmed, and future studies should further define their identity using additional mesangial markers and functional analyses. Third, the present study did not directly assess slit diaphragm integrity. Future studies using slit diaphragm-associated markers, such as nephrin and ZO-1, will be required to determine whether this coculture system supports more advanced podocyte structural maturation or whether doxorubicin disrupts slit diaphragm-related structures. In addition, doxorubicin treatment and FITC-dextran permeability assays were performed as separate endpoint analyses; therefore, whether doxorubicin-induced injury directly alters size-dependent permeability remains unclear. Future studies should optimize combined drug-exposure and permeability assays, as prolonged incubation after doxorubicin treatment may confound permeability measurements through cell detachment or nonspecific loss of the cell layer. Finally, the absence of endothelial cells and physiological flow conditions limits the physiological relevance of the permeability assay. Incorporating glomerular endothelial cells and fluidic stimulation may enable the development of a more complete glomerulus-like model.

In conclusion, co-culture with embryonic rSPCs enhanced podocyte-associated marker expression and promoted podocyte-like characteristics in a pore-size-dependent manner. The co-culture model maintained size-dependent dextran permeability and exhibited a distinct response to doxorubicin-induced injury compared with mono-culture. Although further refinement is required to reproduce the mature glomerular filtration barrier, this membrane-based HSMP–rSPC co-culture system provides a platform for investigating stromal–podocyte interactions.

## CRediT authorship contribution statement

**Tien-Hsiang Tang:** Writing – original draft, Methodology, Investigation, Formal analysis, Data curation, Conceptualization. **Kotaro Doi:** Writing – review & editing, Resources. **Yasuyuki Sakai:** Writing – review & editing, Supervision. **Masaki Nishikawa:** Writing – review & editing, Supervision, Project administration, Funding acquisition.

## Ethics declaration

This study was conducted in accordance with the ARRIVE (Animal Research: Reporting of In Vivo Experiments) guidelines. This study was approved by the University of Tokyo. (Approval No. A2024E024-01)

## Funding

This study was supported by the funds from 10.13039/501100001691JSPS KAKENHI Grants: Grant-in-Aid for Research Activity Start-up (19K23600); Grant-in-Aid for Scientific Research (B) (20H04499, 25K03449); and Grant-in-Aid for Transformative Research Areas (B) (20H05744).

## Declaration of competing interest

The authors declare no competing interests.

## Data Availability

Data will be made available on request.
